# Supplementary material for: RL2 Enhances the Elimination of Breast Cancer Cells by Doxorubicin
Source: Cells. 2023 Dec 6;12(24):2779. doi: 10.3390/cells12242779 (PMC10741759; doi:10.3390/cells12242779)
Supplement: Supplementary file 1 [file cells-12-02779-s001.zip › cells-2659793-supplementary.pdf]

cells MDPI

Supplementary Figures

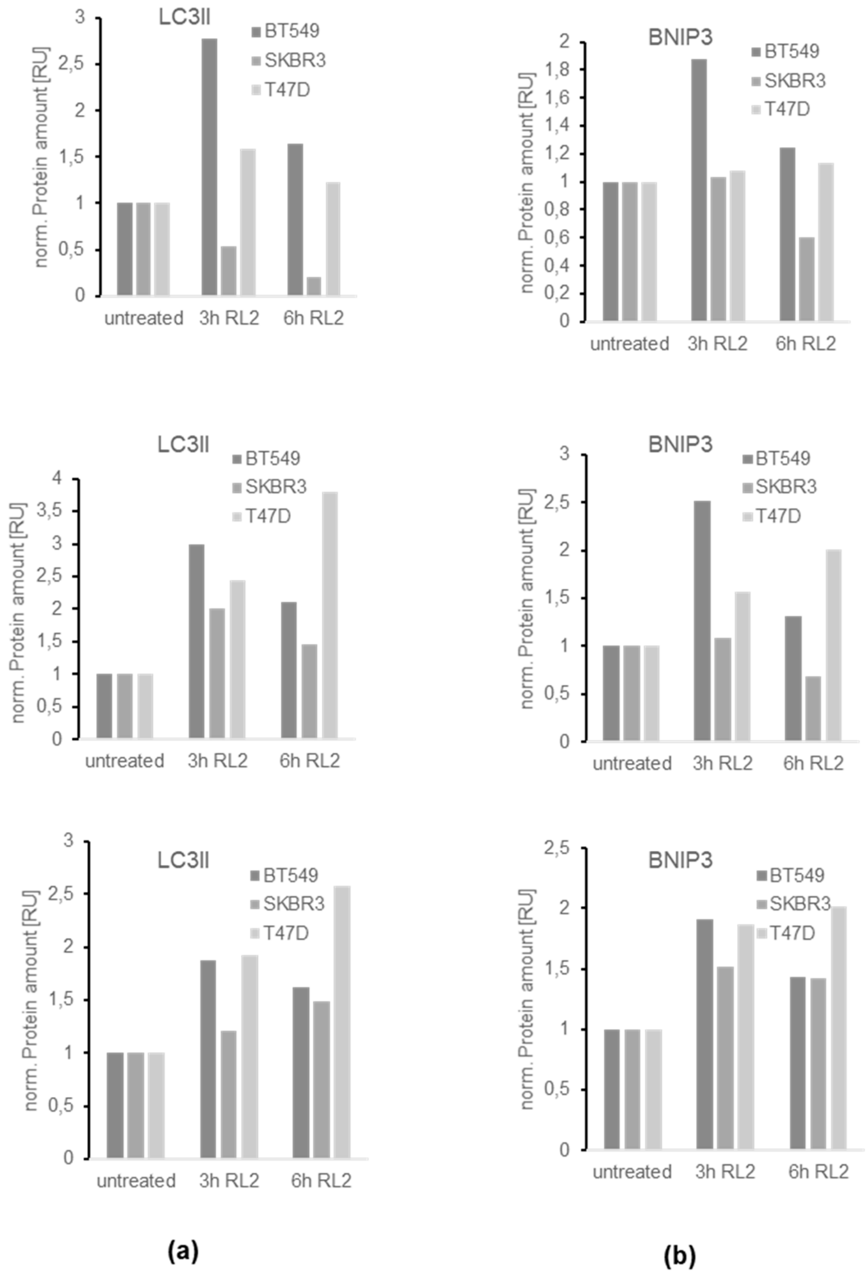

Figure S1. RL2 increases levels of LC3 II and BNIP3 in BT549 and T47D cells: Western Blot quantifications of the signals corresponding to LC3 II (**a**) and BNIP3 (**b**) in Figure 1b.
The quantification of protein band intensities from three independent Western Blot experiments (presented from top to bottom) was performed with Image Lab5.1 (BioRad). The signal intensity was normalized to the loading control signal (actin).

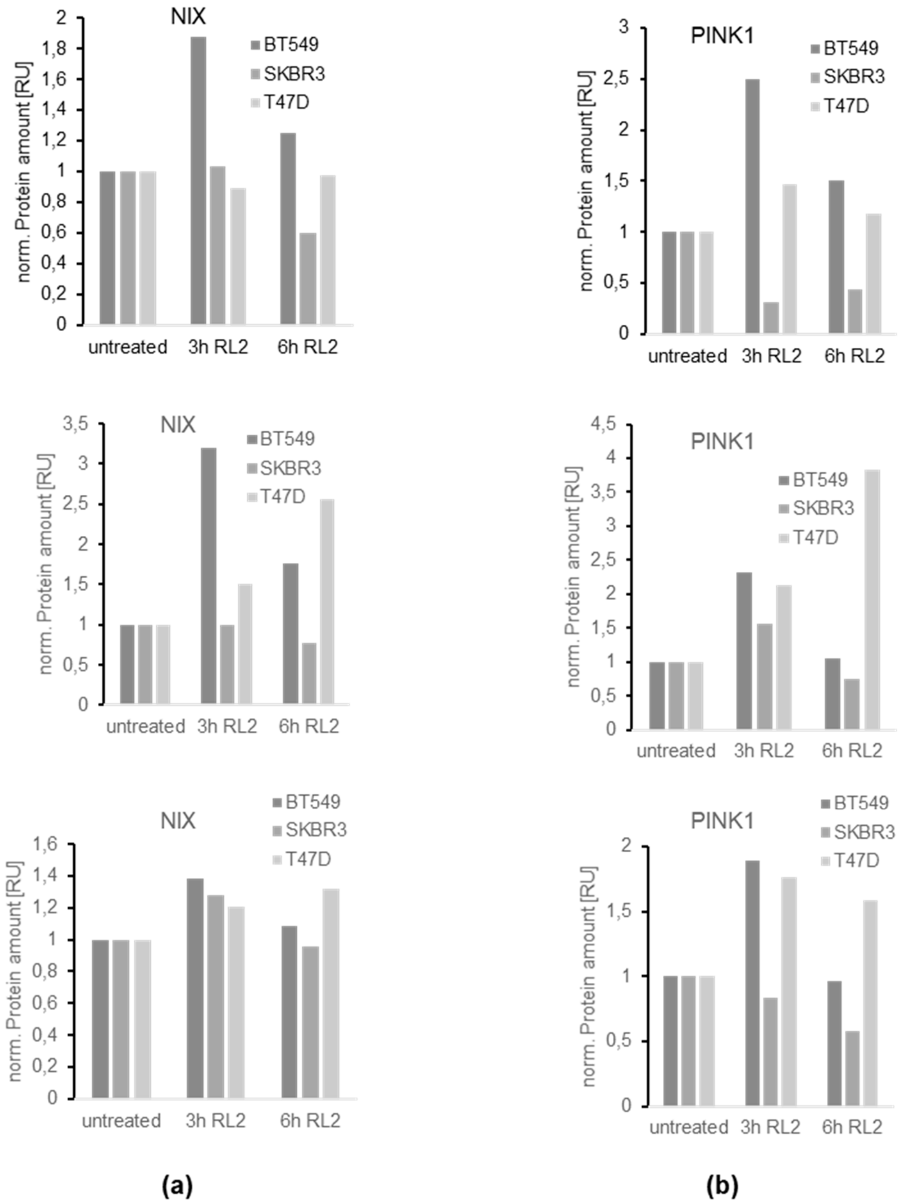

Figure S2. RL2 increases levels of NIX and PINK1 in BT549 cells: Western Blot quantifications of the signals corresponding to NIX (**a**) and PINK1 (**b**) in Figure 1b.

The quantification of protein band intensities from three independent Western Blot experiments was performed with Image Lab5.1 (BioRad). The signal intensity was normalized to the loading control signal (actin).

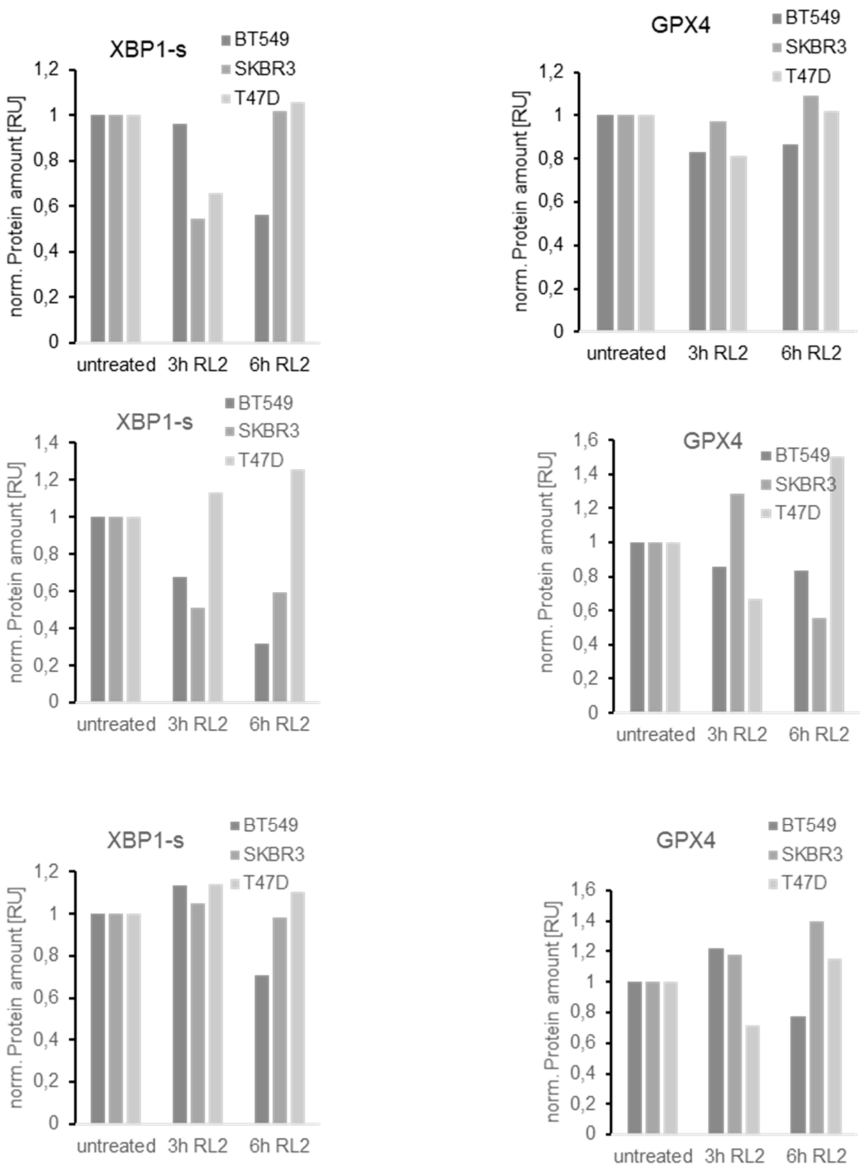

(a) (b)

Figure S3. RL2 has no increasing effect on levels of XBP1-s and GPX4 in different cell lines: Western Blot quantifications of the signals corresponding to XBP1-s (**a**) and GPX4 (**b**) in Figure 1b.

The quantification of protein band intensities from three independent Western Blot experiments was performed with Image Lab5.1 (BioRad). The signal intensity was normalized to the loading control signal (actin).

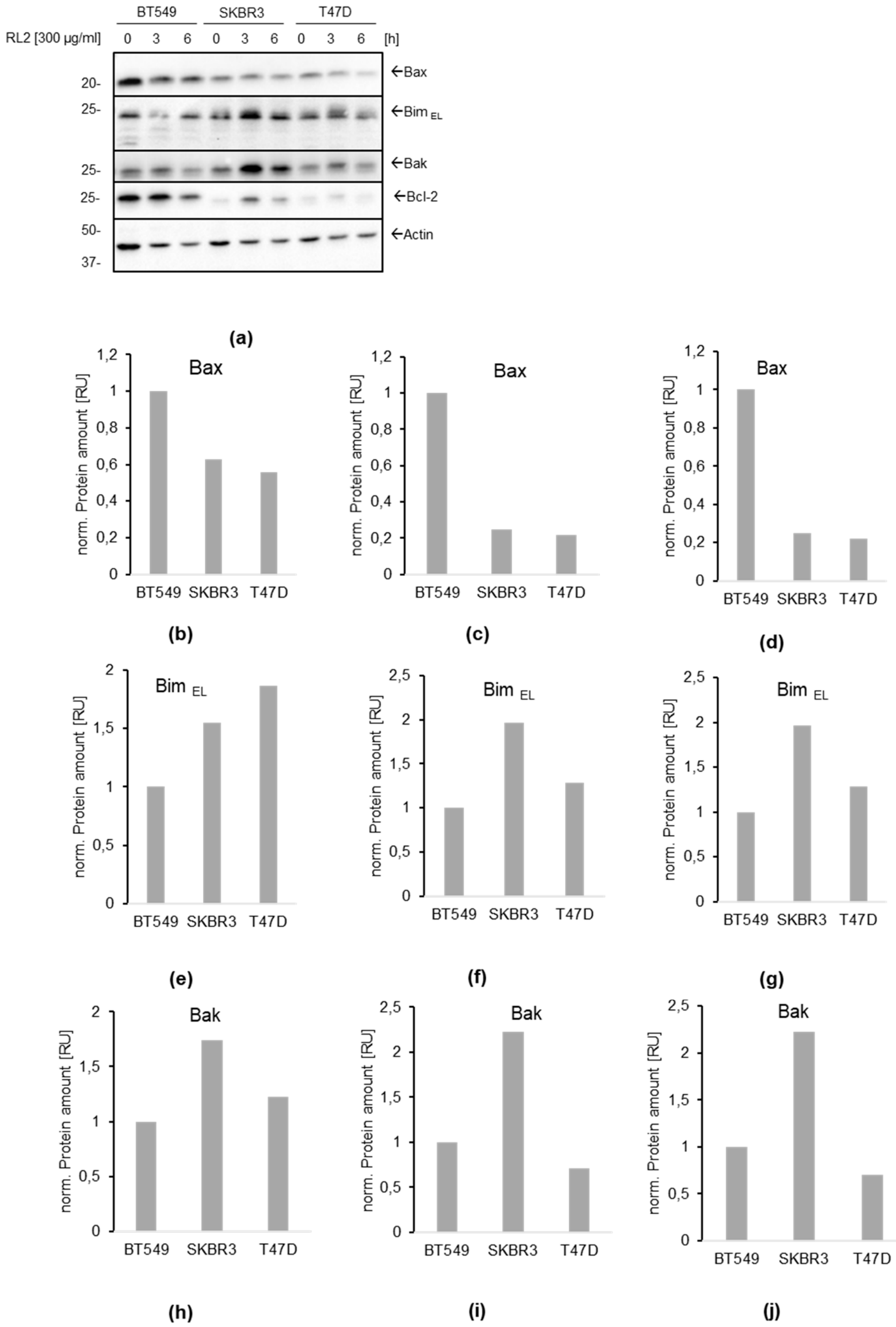

Figure S4. Profiling pro-apoptotic Bcl-2 family members in three different breast cancer cell lines: (**a**) BT549, T47D and SKBR3 cells were treated with 300 µg/mL RL2 for 3 or 6 hours (h) or left untreated. Western Blot analysis of the indicated proteins. One representative experiment out of three independent ones is shown. Western Blot quantifications of the signals corresponding to the lysates of the untreated cells for Bax (**b-d**), Bim EL (**e-g**) and Bak (**h-j**) in Figure S5a.

The quantification of protein band intensities from three independent Western Blot experiments was performed with Image Lab5.1 (BioRad). The signal intensity was normalized to the loading control signal (actin).

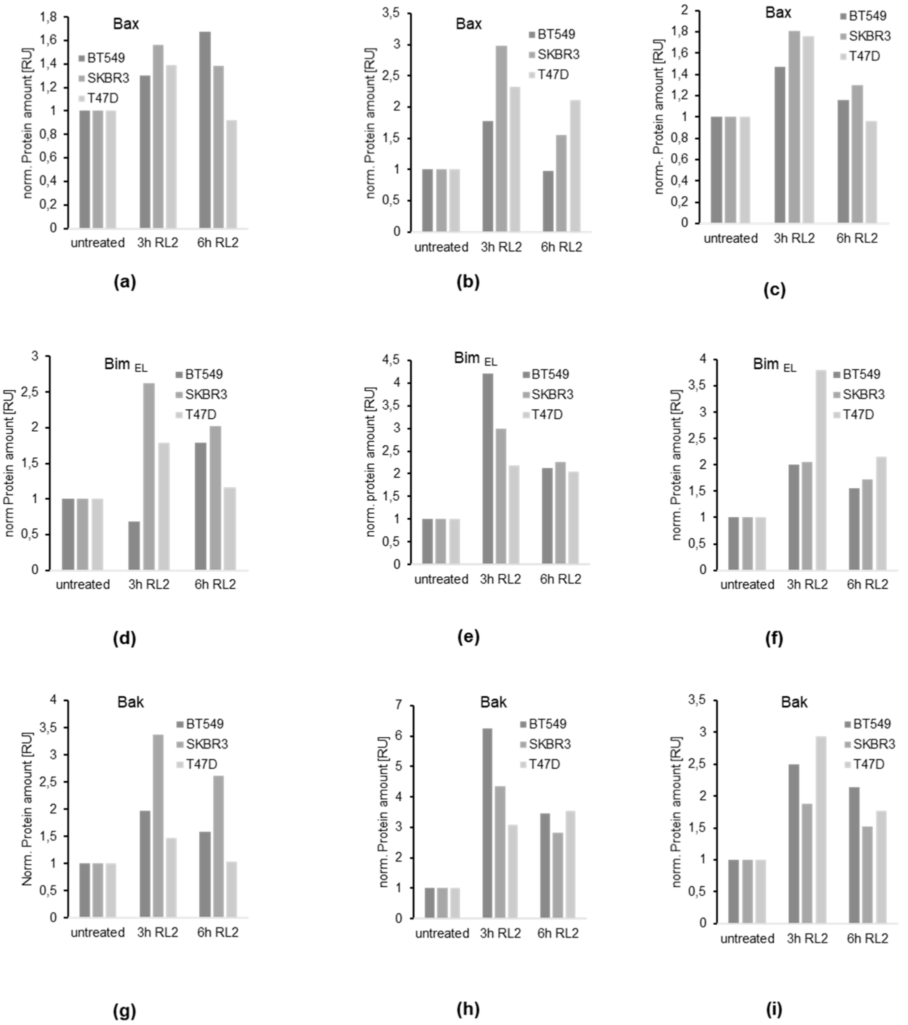

Figure S5. RL2 treatment leads to opposite effects on the expression of pro-apoptotic Bcl-2 family members in different cell lines: Western Blot quantifications of the signals corresponding to lysates of untreated cells for Bax (**a-c**), Bim EL (**d-f**) and Bak (**g-i**) in Figure S5a.

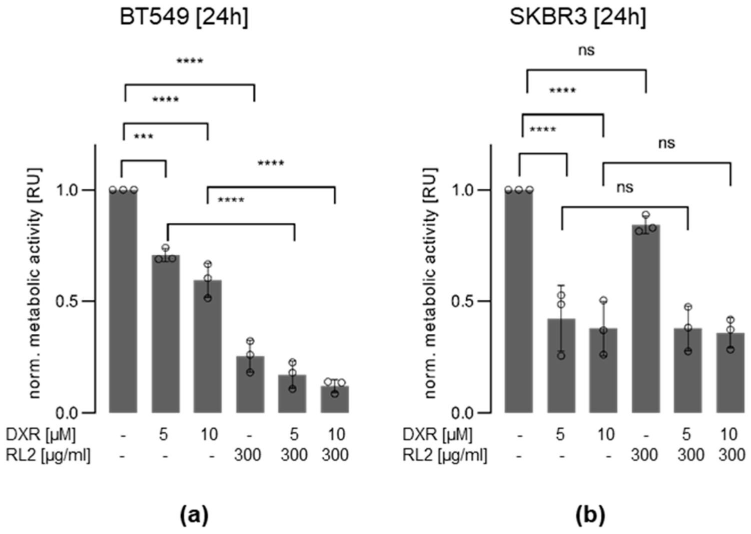

Figure S6. RL2/ DXR co-treatment leads to stronger decrease of metabolic activity in BT549 cells: (**a**) BT549 and (**b**) SKBR3 cells were treated with RL2, DXR or both for the indicated concentrations and for 24 hours (h). Cell viability was captured by measuring metabolic activity using RealTime-Glo™ MT cell Viability Assay Mean and standard deviation are shown for three independent experiments. Statistical analysis was carried out by ordinary ONE-WAY ANOVA with Tukey-test (ns= not significant;** significant; $p<0.002$; *** significant; $p<0.0002$; **** significant; $p<0.0001$).

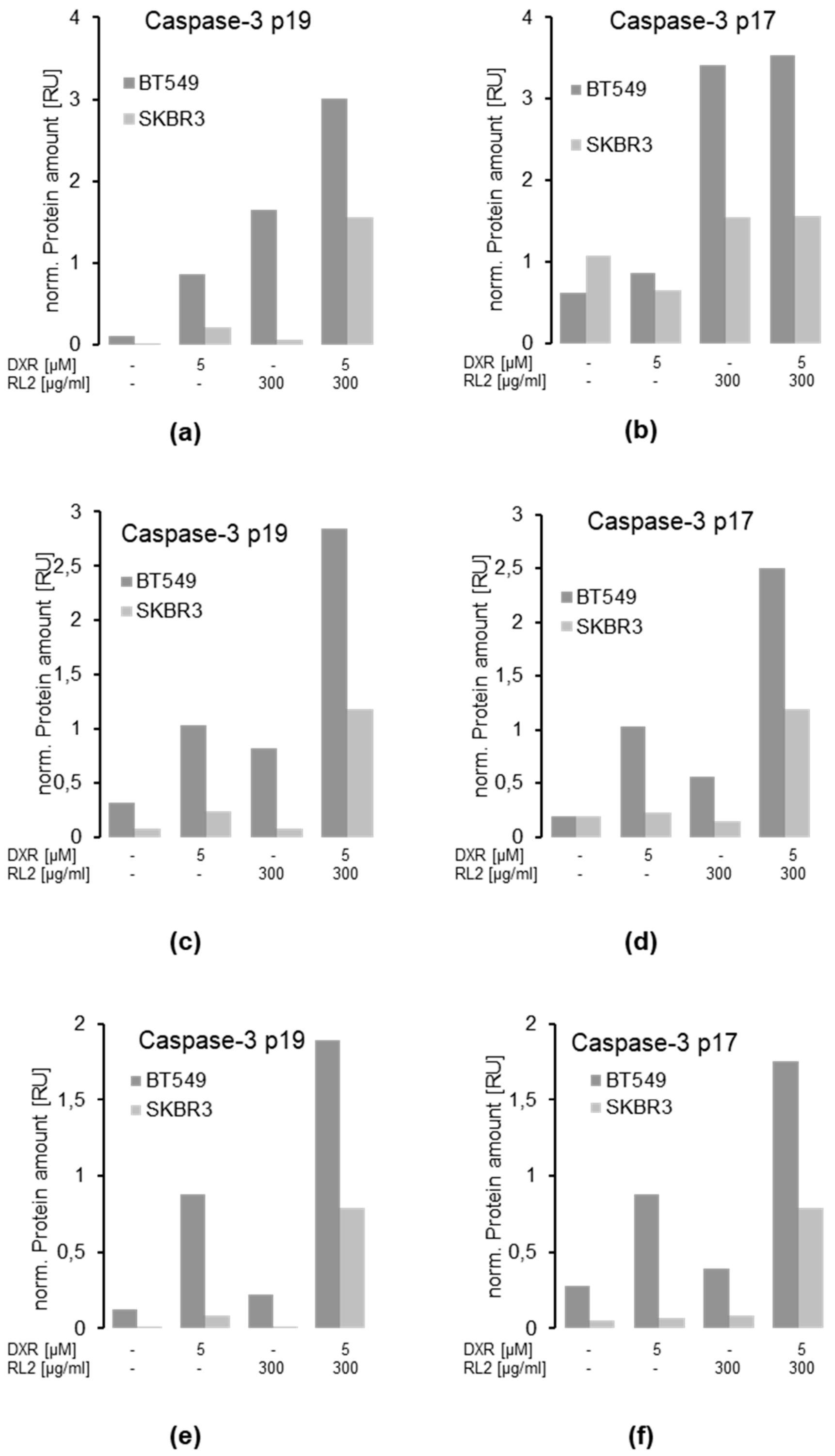

Figure S7. RL2 leads to an increase of Caspase-3 p19 and p17 in RL2-resistant and sensitive cells: Western Blot quantifications of the signals corresponding to Caspase-3 p19 (**a-c**) and Caspase-3 p17 (**d-f**) in Figure 3d. The quantification of protein band intensities from three independent Western Blot experiments was performed with Image Lab5.1 (BioRad). The signal intensity was normalized to the loading control signal (actin).

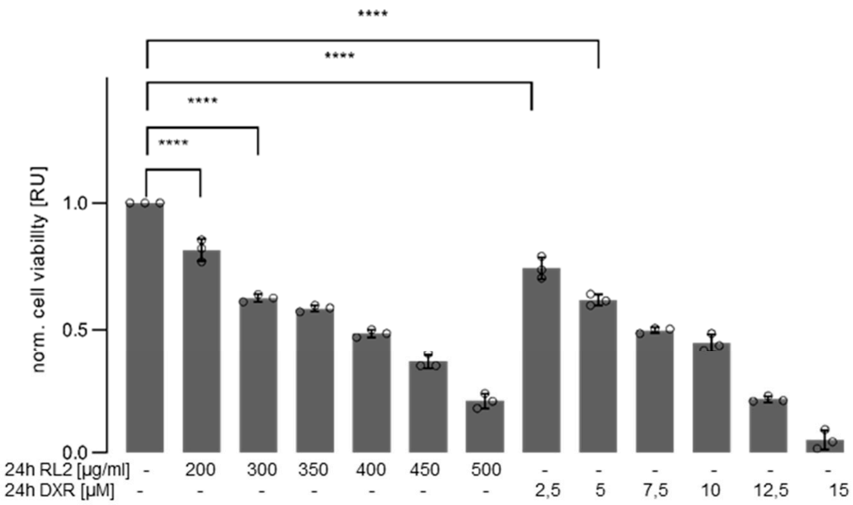

(a)

CI < 1 → synergistic
CI = 1 → additive
CI > 1 = subadditive / antagonistic

(b)

	1,5 µM DXR	5 µM DXR	10 µM DXR
300 µg/ml RL2	1,05	1,59	2,36

(c)

	0,5 µM DXR	1 µM DXR	1,5 µM DXR	2 µM DXR	2,5 µM DXR
50 µg/ml RL2	0,28	0,42	0,56	0,70	0,85
100 µg/ml RL2	0,42	0,56	0,70	0,84	0,98
200 µg/ml RL2	0,69	0,83	0,97	1,11	1,26
300 µg/ml RL2	0,96	1,10	1,25	1,39	1,53
400 µg/ml RL2	1,23	1,38	1,52	1,66	1,80

(d)

	0,5 µM DXR	1µM DXR	2,5 µM DXR
50µg/ml RL2	0,30	0,66	0,63
100µg/ml RL2	0,29	0,53	0,69
200µg/ml RL2	0,42	0,62	0,74
300µg/ml RL2	0,65	0,80	0,89

(e)

Figure S8. Lower concentrations of RL2 and DXR lead to synergetic effects: Calculating synergistic effects and CI of DXR and RL2 treatments.
(**a**) BT549 cells were treated with RL2 or DXR with the indicated concentrations for 24 hours (h). Cell viability was captured by measuring ATP levels using CellTiter-Glo® Luminescent Cell Viability Assay. Mean and standard deviation are shown for three independent experiments. Statistical analysis was carried out by ordinary ONE-WAY ANOVA with Tukey-test (ns= not significant;** significant; p<0.002; *** significant; p<0.0002; **** significant; p<0.0001). Abbreviations: h, hours. Calculation of IC50 (Figure 4a) depending on this Assay. (**b**) Defining of CI according to Loewe Additivity formula. Calculated CI <1, synergistic; CI= 1, additive; CI> subadditive/ antagonistic. (**c**) Calculating synergistic, additive or antagonistic effects of used RL2 and DXR combinatorial treatments from Figure 2. Shown are the CI of the mean of three independent ATP assays. (**d**) Calculating synergistic,

additive or antagonistic effects of potential concentrations of RL2 and DXR combination. (**e**) Calculating CI using response additivity and synergistic, additive or antagonistic effects of Figure 4d. Shown are the CI of the mean of three independent ATP-Assay. (**b-e**) green area = synergistic effects; yellow area = additive effects; red area = subadditive / antagonistic effects
